# Effect of different dialysis duration on the prognosis of peritoneal dialysis-associated peritonitis: a single-center, retrospective study

**DOI:** 10.1080/0886022X.2023.2177496

**Published:** 2023-02-14

**Authors:** Qichen Liang, Huiping Zhao, Bei Wu, Qingyu Niu, Lixia Lu, Jie Qiao, Chuncui Men, Yuting He, Xinxin Chu, Li Zuo, Mei Wang

**Affiliations:** Department of Nephrology, Peking University People’s Hospital, Beijing, China

**Keywords:** Peritoneal dialysis, peritoneal dialysis-associated peritonitis, treatment failure, dialysis duration

## Abstract

**Background:**

Peritoneal dialysis (PD)-associated peritonitis is a serious complication observed in peritoneal dialysis patients. Herein, we investigated the clinical characteristics and treatment outcomes of PD peritonitis in patients with different PD durations.

**Methods:**

All peritonitis episodes from January 2007 to December 2020 at Peking University People’s hospital PD center were retrospectively analyzed and divided into the long-dialysis duration (≥60 months, LDD) and short-dialysis duration (<60 months, SDD) groups. Clinical characteristics and outcomes were compared between these groups. The risk factors for treatment failure were analyzed using a logistic regression model.

**Results:**

During 14 years, 156 patients had 267 peritonitis episodes. There were 83 (31.1%) peritonitis episodes in the LDD group and 184 (68.9%) in the SDD group. No statistical difference was noted in peritonitis causes and the composition of causative pathogens between the two groups. The hospitalization, treatment failure, and transfer-to-hemodialysis rates, and peritonitis-related mortality were significantly higher in the LDD group than in the SDD group (all *p* < .05). Logistic regression analysis revealed that PD duration was an independent risk factor for PD-associated hospitalization, treatment failure and peritonitis-related death (*p* < .05). The receiver operating characteristic curve analysis results showed that when the cutoff value of PD duration was 5.5 years, the sensitivity of predicting PD peritonitis treatment failure was 51.1%, specificity was 78.8%, and the area under the curve was 0.679 (95% confidence interval: 0.594–0.765, *p* < .001).

**Conclusions:**

PD duration is an independent risk factor for poor prognosis in PD peritonitis. Careful and active attention should be paid to the prevention of peritonitis in PD patients with long PD duration.

## Introduction

Peritoneal dialysis (PD)-associated peritonitis is a common and serious complication of PD; it is the most common reason for PD discontinuation [1]. Severe or recurrent peritonitis can cause changes in the structure and function of the peritoneum. Besides causing decreased peritoneal ultrafiltration, malnutrition, increased volume overload, and hospitalization in patients, severe PD peritonitis leads to patients’ withdrawal from PD and being transferred to hemodialysis and even death. With the recent and continuous improvement of PD-related technologies (such as the twin-bag system) and patient management level, the incidence of PD peritonitis has decreased each year; however, there are still great differences among different countries and PD centers, and the pathogen of peritonitis also keeps changing [2]. With the extension of the survival time of PD patients, the clinical characteristics of PD peritonitis in patients with long PD duration are being noticed increasingly. In a previous study, the cutoff value was taken as 36 months for analyzing the clinical characteristics and treatment outcome of PD peritonitis in patients with long PD duration; the results showed that patients with PD peritonitis and longer PD duration had a higher risk of gram-negative bacteria infection and that longer PD duration was an independent risk factor for catheter removal and treatment failure of peritonitis [3].

The cutoff value of 36 months [3] of long PD duration does not fully reflect the overall situation of the current prolonged dialysis duration of PD patients. Thus, in this single-center, retrospective study, we aimed to explore the clinical characteristics and prognosis of PD peritonitis in patients with different dialysis durations using 5 years as the cutoff value to provide evidence for clinical prevention and treatment of PD peritonitis.

## Materials and methods

### Subjects

In this single-center, retrospective study, we included all PD peritonitis episodes that occurred at Peking University People’s hospital PD center from 1 January 2007, to 31 December 2020. Dialysis modalities included intermittent PD (IPD), continuous ambulatory PD (CAPD), and automated peritoneal dialysis (APD). The daily dialysate dose of IPD and CAPD patients was >6 L, the fluid was exchanged 3–5 times each day, and the daily dialysate dose for APD patients was 8–10 L. Conventional glucose-based, lactate-buffered PD solutions (Ultrabag; Baxter Healthcare, Guangzhou, China) were used for all patients.

This study was approved by the Ethics Committee of Peking University People’s Hospital (Ethical approval number: 2021PHB085-001). As this study was a retrospective observational study without any intervention, informed consent was exempted by the Ethics Committee.

### Diagnosis and treatment process of peritonitis

We referred to the guideline of the International Society for Peritoneal Dialysis (ISPD) on PD peritonitis [4] for the diagnostic criteria of PD-associated peritonitis. The patients were diagnosed with PD peritonitis if they met at least two of the following criteria: (1) clinical features consistent with peritonitis, i.e. abdominal pain and/or cloudy dialysis effluent; (2) a dialysis effluent white cell count of >100/μL or >0.1 × 10^9^/L (after a dwell time of at least 2 h), with >50% polymorphonuclear leukocytes; or (3) positive dialysis effluent culture. Relapsing peritonitis refers to peritonitis that occurs within 4 weeks of therapy completion of a prior episode with the same organism or one sterile episode. Peritonitis-related death was defined as death with active peritonitis or within 4 weeks of a peritonitis episode or any death during hospitalization for a peritonitis episode. Treatment failure was defined as transfer to hemodialysis, peritonitis-related death, or relapse.

We have developed our own center’s management process and continue to follow it in daily treatment. Patients with suspected peritonitis are required to immediately come to the hospital; then, dialysis effluent should be drained, carefully inspected, and sent for cell count with differential, Gram stain, and culture. If there is an exit-site infection, the bacterial culture of the discharge should be simultaneously performed.

Once the diagnostic investigations have been completed, empirical antibiotics should be started. Empirical antibiotic therapy must act on both gram-positive and gram-negative bacteria. According to the drug susceptibility results of causative organisms from our center, the initial empirical treatment included a combination of a first-generation cephalosporin and a third-generation cephalosporin administered intraperitoneally as an intermittent dose: ceftazidime and cefazolin 1.0 g, respectively, were added to 2 L peritoneal dialysate once a day, dwelling for at least 6 h. We use vancomycin for gram-positive bacteria in patients allergic to first-generation cephalosporins. On the 3rd day and the 5th day, cell counts were performed to observe the therapeutic effect. Once the bacterial culture results were reported, sensitive antibiotics were selected according to the drug susceptibility results. If peritonitis was not controlled after appropriate antibiotic treatment for 5 days, refractory peritonitis was diagnosed. For refractory or fungal peritonitis, catheters should be removed in time.

### Data collection

The baseline characteristics of all patients, including age and sex as well as the primary disease of end-stage kidney disease (ESKD), diabetes mellitus, PD duration, and PD prescription, were recorded. Height and body weight were also recorded; body mass index (BMI) was calculated. Clinical and biochemical data, including the time from the first presentation to a hospital facility (whether delayed treatment), cause of peritonitis, clinical symptoms and signs, causative organisms, dialysate white blood cell counts (dWBC) on day 0 and day 3, and whether outpatient or inpatient treatment, were collected at the time of PD peritonitis. We also recorded the latest biochemical data, including hemoglobin level; serum albumin, potassium, and phosphate levels; and PD adequacy indices [renal and peritoneal Kt/V (urea clearance index)], within 1 month before the occurrence of peritonitis.

### Statistical analysis

Categorical variables were expressed as numbers and percentages, and continuous variables were expressed as mean ± standard deviation (SD). Continuous variables were interpolated by mean or median in line with the type of distribution, whereas categorical variables were interpolated by mode. All peritonitis episodes were divided into the long-dialysis duration (≥60 months, LDD) and the short-dialysis duration (<60 months, SDD) groups. Differences between the groups were analyzed using the chi-square or Fisher’s exact test for categorical data and the Student t-test or Mann–Whitney U-test for continuous data. Univariate analysis was used for the preliminary exploration of variables; *p* < .05 indicated a potential predictor. Binary logistic regression was used to analyze the risk factors for poor prognosis of peritonitis; the enter method was applied for binary logistic regression analysis. Multicollinearity was assessed by checking the Variance Inflation Factor on a multiple linear regression model. The receiver operating characteristic (ROC) curve was used to analyze the cutoff value of PD duration for treatment failure. Additional sensitivity analyses were conducted: (1) excluding culture-negative peritonitis; (2) including only first-time peritonitis.

A two-tailed *p*-value of <.05 was considered statistically significant. All statistical analyses were performed using the SPSS software (version 26.0: SPSS, Chicago, IL, USA).

## Results

### Study population and general characteristics of 267 peritonitis episodes

**Figure 1.**
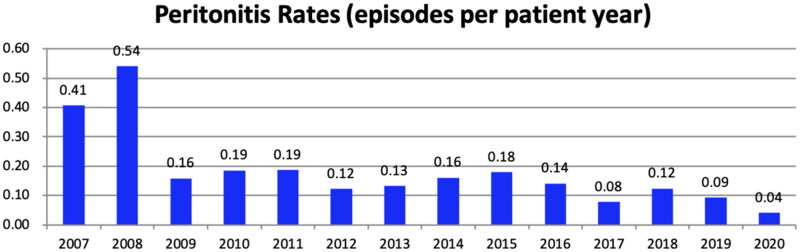
Peritonitis rates by year in our center from 2007 to 2020.

Over 14 years, 267 episodes of peritonitis occurred in 156 patients. Among these patients, 93 (59.6%) occurred one episode, 39 (25.0%) occurred two episodes, and 24 (15.4%) occurred three or more episodes of peritonitis. The annual peritonitis rates in our center are listed in Figure 1 which have been lower than 0.10 episodes per year in recent years. Among all 267 episodes, 147 (55.1%) occurred in female; the age of onset was 63 (56–72) years, and the median PD duration was 40 (19–68) months. The primary disease was chronic glomerulonephritis (CGN) in 97 (36.3%) cases, diabetic nephropathy in 90 (33.7%) cases, hypertensive nephropathy in 40 (15.0%) cases, chronic tubulointerstitial nephropathy in 29 (10.9%) cases. There were 134 (50.2%) patients with diabetes.

### Comparison of clinical data and prognosis between the groups

Of all peritonitis episodes, there were 83 (31.1%) cases in the LDD group and 184 (68.9%) in the SDD group. The median PD duration in the LDD group was 80 (71–106) months and that in the SDD group was 26 (11–42) months. Patients in the LDD group had a lower incidence of diabetes (*p* < .001) and lower hemoglobin level (before peritonitis) (*p* = .024) than those in the SDD group; the LDD group had a higher proportion of females than the SDD group (*p* < .001) (Table 1).

**Table 1.** General data and the latest biochemical data before peritonitis.

Variable	All peritonitis (*n* = 267)	SDD group (*n* = 184)	LDD group (*n* = 83)	*p*-value
Female, *n* (%)	147 (55.1)	88 (47.8)	59 (71.1)	<.001**
Age (years)	63 (56, 72)	64 (56, 72)	63 (56, 73)	.795
PD duration (months)	40 (19, 68)	26 (11, 42)	80 (71, 106)	<.001**
Primary disease (%)				
Chronic glomerulonephritis	97 (36.3)	47 (25.5)	50 (60.2)	<.001**
Diabetic nephropathy	90 (33.7)	78 (42.4)	12 (14.5)	<.001**
Hypertensive nephropathy	40 (15.0)	29 (15.8)	11 (13.3)	.595
Chronic tubulointerstitial nephropathy	29 (10.9)	19 (10.3)	10 (12.0)	.676
Obstructive nephropathy	1 (0.4)	1 (0.5)	0 (0)	1.000
Other	10 (3.7)	10 (5.4)	0 (0)	.034*
Diabetes (%)	134 (50.2)	109 (59.2)	25 (30.1)	<.001**
CAPD, n (%)	241 (90.3)	163 (88.6)	78 (94.0)	.169
BMI (kg/m^2^)	22.60 (20.62, 24.78)	22.63 (20.81, 24.91)	22.04 (20.43, 24.22)	.446
K (mmol/L)	4.10 (3.68, 4.61)	4.15 (3.68, 4.63)	4.06 (3.67, 4.51)	.164
Alb (g/L)	35.70 (31.80, 38.03)	36.10 (31.60, 38.60)	34.70 (32.00, 37.30)	.118
Hb (g/L)	114 (102, 121)	114 (104, 122)	112 (98, 119)	.024*
P (mmol/L)	1.46 ± 0.46	1.46 ± 0.42	1.47 ± 0.53	.927
Kt/V urea (/week)	1.82 (1.59, 2.11)	1.80 (1.56, 2.13)	1.87 (1.60, 2.06)	.556

SDD: short-dialysis duration; LDD: long-dialysis duration; PD: peritoneal dialysis; CAPD: continuous ambulatory peritoneal dialysis; BMI: body mass index; Alb: serum albumin levels; Hb: hemoglobin; P: phosphate; K: Potassium.

* *p* < 0.05 **p < 0.01.

Table 2 shows the PD peritonitis-related characteristics of the two groups. The most common causes of peritonitis in the two groups were exchange-related contamination and enteric infection; however, there was no statistical difference between the two groups. The number of dWBC on day 3 in the LDD group was significantly higher than that in the SDD group (*p* = .003), whereas the serum albumin level during peritonitis was significantly lower in the LDD group than that in the SDD group (*p* = .026). The culture-positive rate of all peritonitis was 77.2%, and the causative pathogens were mainly gram-positive bacteria (47.6%). No statistical difference was noted in the composition of causative pathogens between the two groups. The hospitalization rate, treatment failure rate, transfer-to-hemodialysis rate, and peritonitis-related mortality in the LDD group were significantly higher than those in the SDD group (all *p* < .05) (Table 3).

**Table 2.** Comparison of clinical data of the peritonitis in different PD duration groups.

Variable	All peritonitis (*n* = 267)	SDD group (*n* = 184)	LDD group (*n* = 83)	*p*-value
Cause of peritonitis (%)				
Exchange-related contamination	118 (44.2)	84 (45.7)	34 (41.0)	.475
Enteric infection	97 (36.3)	62 (33.7)	35 (42.2)	.183
Systemic infection	20 (7.5)	16 (8.7)	4 (4.8)	.265
Catheter-related infection	2 (0.7)	1 (0.5)	1 (1.2)	.526
Malnutrition	5 (1.9)	3 (1.6)	2 (2.4)	.648
Causes unknown	22 (8.2)	15 (8.2)	7 (8.4)	.938
Recent usage of antibiotics	3 (1.1)	3 (1.6)	0	.555
dWBC on day0[cells/uL, M (1/4, 3/4)]	1790 (600, 4000)	1400 (480, 3598)	2313 (890, 4674)	.043*
dWBC on day3[cells/uL, M (1/4, 3/4)]	79 (10, 458)	58 (9, 261)	180 (33, 1020)	.003**
Patients with delayed treatment (%)	97 (36.3)	69 (37.5)	28 (33.7)	.554
Fever (%)	84 (31.5)	59 (32.1)	25 (30.1)	.751
Abdominal pain (%)	217 (81.3)	154 (83.7)	63 (75.9)	.131
Blood leukocyte count [×10^9^/L, M (1/4, 3/4)]	7.58 (6.35, 9.42)	7.52 (6.39, 9.32)	7.59 (6.13, 10.28)	.820
Serum alb (g/L)	33.15 (30.20, 37.15)	33.80 (30.30, 37.70)	32.10 (29.30, 35.50)	.026*
Causative organisms				
Gram-positive pathogens, *n* (%)	127 (47.6)	85 (46.2)	42 (50.6)	.505
Coagulase-negative	60 (22.5)	44 (23.9)	16 (19.3)	.401
Staphylococcus aureus	15 (5.6)	10 (5.4)	5 (6.0)	1.000
Streptococci/Enterococci	40 (15.0)	25 (13.5)	15 (18.0)	.342
Other gram-positive	12 (4.5)	6 (3.3)	6 (7.2)	.259
Gram-negative pathogens, *n* (%)	61 (22.8)	43 (23.4)	18 (21.7)	.762
Escherichia coli	30 (11.2)	22 (12.0)	8 (9.6)	.579
Pseudomonas aeruginosa	4 (1.5)	3 (1.6)	1 (1.2)	1.000
Other gram-negative	27 (10.1)	18 (9.8)	9 (10.8)	.790
Other pathogens, *n* (%)	18 (6.7)	13 (7.1)	5 (6.0)	.753
Fungi	4 (1.5)	4 (2.2)	0	.314
Mycobacterium	2 (0.7)	2 (1.1)	0	1.000
Polymicrobial infection	12 (4.5)	7 (3.8)	5 (6.0)	.623
Culture-negative, *n* (%)	61 (22.8)	43 (23.4)	18 (21.7)	.762

SDD: short-dialysis duration; LDD: long-dialysis duration; PD: peritoneal dialysis; Alb: serum albumin levels; dWBC: dialysate white blood cell counts.

*p < 0.05; **p < 0.01.

**Table 3.** Comparison of prognosis of peritonitis at different PD duration groups.

Prognosis, *n* (%)	All peritonitis (*n* = 267)	SDD group (*n* = 184)	LDD group (*n* = 83)	*p*-value
Hospitalization	70 (26.2)	36 (19.6)	34 (41.0)	.001**
Treatment outcome				
Cure	222 (83.1)	163 (88.6)	59 (71.1)	.001**
Transfer to HD	23 (8.6)	11 (6.0)	12 (14.5)	.022*
Death	20 (7.5)	9 (4.9)	11 (13.3)	.016*
Relapse	2 (0.7)	1 (0.5)	1 (1.2)	.526

HD: hemodialysis.

* *p* < .05 **p < .01.

### Independent factors associated with peritonitis prognosis

In our study, univariate analysis revealed that PD duration, serum albumin (before peritonitis), phosphate (before peritonitis), serum albumin (at onset), dWBC on day 0, dWBC on day 3, blood leukocyte count were strongly associated with treatment failure of peritonitis (Supplemental Table S1). In the univariate logistic analysis (Model 1), we found that PD duration was a risk factor for treatment failure [odds ratio (OR): 1.228, 95% confidence interval (CI): 1.105–1.364, *p* < .001]. Multivariate logistic regression Model 2 showed that after age and sex were adjusted, PD duration was an independent risk factor for treatment failure of peritonitis (OR: 1.258, 95% CI: 1.124–1.407, *p* < .001). After adjustment for age, year of peritonitis, diabetes, serum albumin before and after peritonitis and causative organisms in the Model 3, the PD duration (OR: 1.449, 95% CI: 1.024–2.050, *p* = .036), white blood cell count at diagnosis (OR: 1.413, 95% CI: 1.119–1.785, *p* = .004), dWBC on day 3 (OR: 1.173, 95% CI: 1.063–1.295, *p* = .002) were independent risk factors for treatment failure, whereas female (OR: 0.134, 95% CI: 0.021–0.846, *p* = .033) and serum phosphorus level (before peritonitis) were protective factors (OR: 0.069, 95% CI: 0.007–0.671, *p* = .021). The results of the models with and without adjustments are shown in Table 4. Meanwhile, the PD duration was also an independent risk factor for peritonitis hospitalization (OR: 1.163, 95% CI: 1.009–1.341, *p* = .038) and peritonitis-related death (OR: 1.815, 95% CI: 1.015–3.244, *p* = .044).

**Table 4.** Logistic regression analysis of risk factors associated with treatment failure of PD peritonitis.

Variables	OR	95%CI	*p*-value
*Model 1: no adjustment*			
PD duration (per 1 year increase)	1.228	1.105 ∼ 1.364	<.001**
*Model 2: adjusted for age and sex*			
Age (per 1 year increase)	1.018	0.989 ∼ 1.048	.230
Sex (female)	0.611	0.302 ∼ 1.238	.172
PD duration (per 1 year increase)	1.258	1.124 ∼ 1.407	<.001**
*Model 3: multivariable adjusted model ^a^*			
Age (per 1 year increase)	0.983	0.915 ∼ 1.055	.630
Sex (female)	0.134	0.021 ∼ 0.846	.033*
PD duration (per 1 year increase)	1.449	1.024 ∼ 2.050	.036*
Year of peritonitis^b^			
2007–2010	referent	referent	–
2011–2015	2.361	0.035 ∼ 158.136	.689
2016–2020	7.304	0.096 ∼ 556.203	.368
Serum Albumin (per 1 g/L increase)(before peritonitis)	0.854	0.689 ∼ 1.060	.153
Phosphate (per 1 mmol/L increase)(before peritonitis)	0.069	0.007 ∼ 0.671	.021*
Serum Albumin (per 1 g/L increase)	1.093	0.912 ∼ 1.310	.335
dWBC on day 3 (per 100cells/uL increase)	1.173	1.063 ∼ 1.295	.002**
Blood leukocyte count (per 10^9^/L increase)	1.413	1.119 ∼ 1.785	.004**
Causative organisms^c^			
Gram-positive peritonitis	referent	referent	–
Gram-negative peritonitis	6.111	0.785 ∼ 47.553	.084
Other pathogens	16.889	1.656 ∼ 172.234	.017*
Culture-negative peritonitis	6.144	0.599 ∼ 63.045	.126

PD: peritoneal dialysis; PDAP: peritoneal dialysis-associated peritonitis; OR: odds ratio; CI: confidence interval; PD: peritoneal dialysis; dWBC: dialysate white blood cell counts.

* *p* < .05 **p < .01.

^a^Adjusted for age, sex, PD duration, year of peritonitis, serum albumin before and after peritonitis, phosphate before peritonitis, dWBC on day 3, blood leukocyte count and causative organisms.

^b^ Compared to 2007–2010.

^c^Compared to gram-positive peritonitis.

### PD duration predicted the risk of treatment failure of PD peritonitis

**Figure 2.**
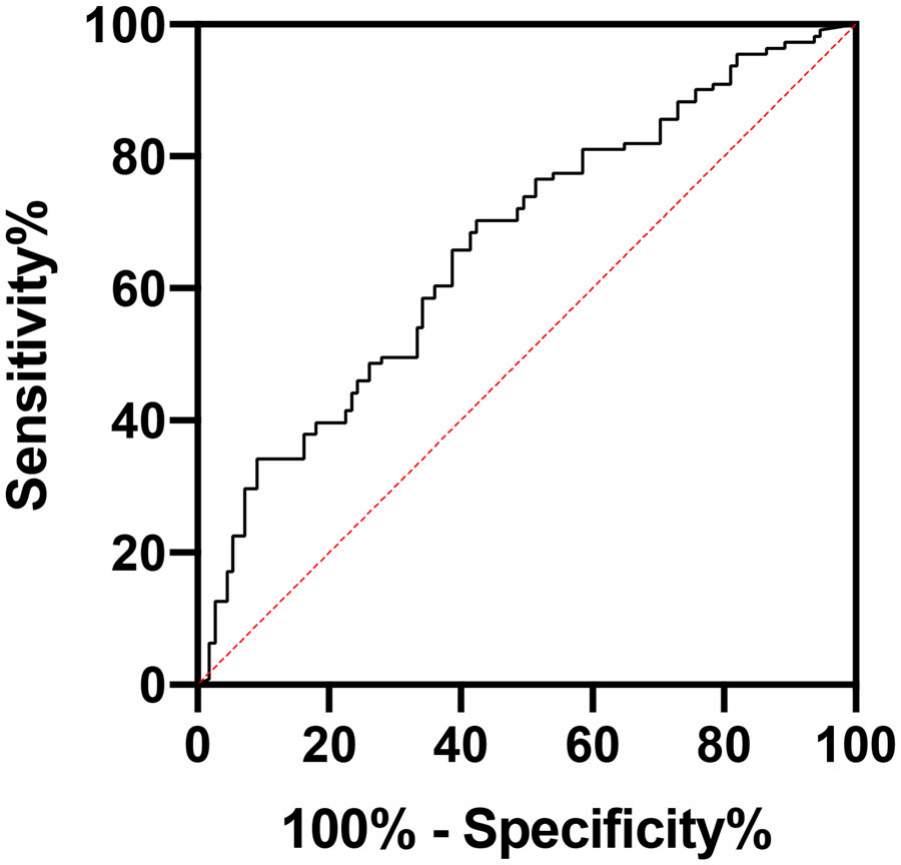
ROC curve of using PD duration to predict PD peritonitis treatment failure.

The results of the ROC curve analysis showed that when the PD duration was defined as 5.5 years, the sensitivity of predicting PD peritonitis treatment failure was 51.1%, the specificity was 78.8%, and the area under the curve was 0.679 (95% CI: 0.594–0.765, *p* < .001) (Figure 2).

## Discussion

In recent years, the PD duration of maintenance PD patients has gradually increased [5]. Regardless of the continuous improvement of PD technology and patient management level, PD peritonitis is still an important cause of technical failure and even death of PD patients [6]. In the present study, by analyzing the clinical data of 267 cases of peritonitis in 156 patients at our center over 14 years, we analyzed the clinical characteristics and prognosis of peritonitis in different PD duration groups.

The study results showed that the LDD group had a lower incidence of diabetes, lower hemoglobin level (before the peritonitis), and a higher proportion of females than the SDD group. Many studies have found that long PD duration patients have lower rates of diabetes [5,7,8]. This may be due to better survival in PD patients without diabetes. Wu et al. found that women had better survival rates with PD [9], which was consistent with our results. However, the previous study [9] found that the technical survival rate of patients with diabetes was poor which may be related to the different demographic data of the study subjects and the different management of each center.

Though there was no difference in pathogens and causes of peritonitis between the two groups in our study; we observed that the treatment failure rates and mortality rates were higher in the LDD group than in the SDD group. Meanwhile, multivariate regression analysis found that the PD duration was an independent risk factor for peritonitis-associated hospitalization and treatment failure. This is consistent with the results of some previous studies [10–15]. The ROC curve also further demonstrated that a dialysis duration of >5.5 years was a predictor of peritonitis treatment failure. No previous study has compared differences in peritonitis prognosis between patients with a PD duration of ≥5 years and <5 years. Previous studies have reported the clinical characteristics of PD patients with long PD duration [7,8,16,17], however, there are relatively few studies on PD peritonitis in long PD duration patients. These studies were relatively early, limited in sample size, and the definition of long duration was <3 years [10,18]. Krishnan [10] found that the treatment failure rate of peritonitis in patients with a PD duration of >2.4 years was significantly higher than that in patients with a PD duration of <2.4 years (24.4% vs 16.5%, *p* = .05), whereas the median PD duration in our study was 40 months, which is much longer. Troidle et al. [18] found that the catheter removal rate in patients with a PD duration of ≥37 months was significantly lower than that in patients with a PD duration of <12 months and 13–36 months (*p* < .05), and there was no significant difference in mortality. This study was early and had a small sample size, which may explain the different results.

It is speculated that the reasons for the poor prognosis of patients with long PD duration are as follows: On the one hand, with the continuous extension of PD duration, the aseptic awareness of patients gradually weakens, and the compliance with regular follow-up becomes poor, leading to the occurrence of peritonitis. Our study demonstrated that the proportion of exchange-related contamination in the LDD group was slightly lower than that in the SDD group, but it was still a main cause of peritonitis; hence, it is necessary to focus on operation-related retraining. Rikako et al. [13] found that the dialysis duration is an independent risk factor for sign to treatment time of ≥ 24h, suggesting that retraining is very important for patients with long dialysis duration. However, our study found that there was no significant difference in the proportion of delayed treatment between the two groups, but the prognosis of the LDD group was worse, which may be related to the changes in peritoneal morphology and function in long-term dialysis patients. Williams et al. [19] reported a gradual increase in the submesothelial compact zone thickness with prolonged PD duration, suggesting changes in peritoneal morphology, which are assumed to be associated with long-term non-biocompatible dialysate exposure [20,21]. Using low glucose degradation product neutral pH solutions can reduce peritoneal interstitial fibrosis and hyaline angiogenesis, thereby preserving peritoneal function and prolonging technical survival [9,22,23]. In addition, the peritoneal immune defense function in patients with long PD duration was also affected. Studies have found that with the prolongation of PD time, the function of peritoneal macrophages to secrete cytokines is abnormal and the expression of CD15, which indicates the macrophage immaturity, increases [24]. A recent Bragg study [25] compared the peritoneal effluent transcriptome of 20 PD patients with a dialysis duration ≤2 years and 13 patients with a dialysis duration >2 years and found that an increased expression of genes associated with an immune response in patients with longer PD duration and duration of PD treatment were positively correlated with the number of granulocytes and natural killer cells in the peritoneal dialysate. The above studies all suggest that long PD duration causes changes in peritoneal structure and function, resulting in a poor prognosis of PD peritonitis.

Our study found that dWBC on day 3 in peritonitis was an independent risk factor for peritonitis treatment failure, which was consistent with a previous study [14]. A retrospective study by Kai et al. [14] showed that a peritoneal dWBC of >1090/mm^3^ on day 3 was an independent prognostic factor for treatment failure. This suggests that patients with peritonitis who have a poor response to antibiotic therapy after 3 days have a worse prognosis. A clinical risk prediction tool [26] also found that dialysate leukocyte count >1000/mm^3^ on days 3–4 is a predictor for peritonitis – associated treatment failure.

Our study is the first one to use the 5-year cutoff value of PD duration for grouping and found that long PD duration is closely related to poor prognosis in peritonitis. However, our study has some limitations. First, there was bias because it was a retrospective study. A larger population size is needed. Since this was a single-center study, the generalizability of results was compromised. Second, our study only analyzed the short-term prognosis of PD peritonitis and not the long-term prognosis of peritonitis. Moreover, multimorbidity, nutritional status, socioeconomic factors, and strategies for preventing PD-related infection should also be considered.

## Conclusion

In conclusion, long PD duration patients with >60 months had a higher PD peritonitis treatment failure rate and higher mortality rate than other PD patients. Long PD duration is an independent risk factor for poor prognosis in PD peritonitis. Therefore, careful and active attention should be paid to the prevention of peritonitis in PD patients with long PD duration, and biocompatible dialysate should be used as much as possible to preserve peritoneal function.

## Supplementary Material

Supplemental Material
