# Supplementary material for: Effect of different dialysis duration on the prognosis of peritoneal dialysis-associated peritonitis: a single-center, retrospective study
Source: Ren Fail. 2023 Feb 14;45(1):2177496. doi: 10.1080/0886022X.2023.2177496 (PMC9930808; doi:10.1080/0886022X.2023.2177496)
Supplement: Supplemental Material [file IRNF_A_2177496_SM7861.pdf]

Supplementary Table S1. Univariate logistic regression analysis of risk factors associated with treatment failure of PD peritonitis.

	Univariate logistic regression		
Variables	OR	95%CI	P-value
Age (per 1 year increase)	1.017	0.989~1.045	0.236
Sex(female)	0.920	0.484~1.749	0.799
PD duration (per 1 year increase)	1.228	1.105~1.364	<0.001**
Year of peritonitis [a]			
2007-2010	referent	referent	-
2011-2015	2.071	0.662~6.481	0.211
2016-2020	6.971	2.275~21.363	0.001**
Diabetes (yes)	1.045	0.551~1.985	0.892
Serum Albumin (per 1g/L increase) (before peritonitis)	0.899	0.843~0.959	0.001**
Phosphate (per 1mmol/L increase) (before peritonitis)	0.439	0.211~0.916	0.028*
Potassium (per 1 mmol/L increase) (before peritonitis)	0.750	0.457~1.230	0.255
Hemoglobin (per 1 g/L increase) (before peritonitis)	1.008	0.988~1.029	0.428
Kt/V urea (per 1 increase) (before peritonitis)	0.564	0.255~1.248	0.158
Serum Albumin (per 1g/L increase)	0.852	0.788~0.920	<0.001**
dWBC on day 0(per 100cells/uL increase)	1.006	1.001~1.012	0.024*
dWBC on day 3(per 100cells/uL increase)	1.114	1.067~1.163	<0.001**
Blood leukocyte count (per 10^9/L increase)	1.279	1.150~1.433	<0.001**
Hemoglobin (per 1 g/L increase)	0.986	0.963~1.009	0.226
Calcium (per 1mmol/L increase)	0.331	0.066~1.653	0.178
Phosphate (per 1mmol/L increase)	0.526	0.207~1.335	0.176

Information Classification: General

Causative organisms [b] Gram-positive peritonitis	referent	referent	-
Gram-negative peritonitis	4.898	2.097~11.440	<0.001
Other pathogens	11.700	3.790~36.119	<0.001
Culture-negative peritonitis	1.766	0.660~4.728	0.258

PD = peritoneal dialysis; PDAP = peritoneal dialysis-associated peritonitis; OR = odds ratio; CI = confidence interval; PD = peritoneal dialysis; dWBC = dialysate white blood cell counts.

* P<0.05 **P<0.01

a. Compared to 2007-2010.

b. Compared to gram-positive peritonitis.

Information Classification: General

Information Classification: General
